# A Trajectory Planning Method for Autonomous Valet Parking via Solving an Optimal Control Problem

**DOI:** 10.3390/s20226435

**Published:** 2020-11-11

**Authors:** Chen Chen, Bing Wu, Liang Xuan, Jian Chen, Tianxiang Wang, Lijun Qian

**Affiliations:** College of Automotive and Transportation Engineering, Hefei University of Technology, Hefei 230009, China; cclwtg@163.com (C.C.); hfutwubing@126.com (B.W.); xaun865807064@126.com (L.X.); 2017110894@mail.hfut.edu.cn (J.C.); wtx417@163.com (T.W.)

**Keywords:** autonomous parking, trajectory planning, homotopic method, optimal control

## Abstract

In the last decade, research studies on parking planning mainly focused on path planning rather than trajectory planning. The results of trajectory planning are more instructive for a practical parking process. Therefore, this paper proposes a trajectory planning method in which the optimal autonomous valet parking (AVP) trajectory is obtained by solving an optimal control problem. Additionally, a vehicle kinematics model is established with the consideration of dynamic obstacle avoidance and terminal constraints. Then the parking trajectory planning problem is modeled as an optimal control problem, while the parking time and driving distance are set as the cost function. The homotopic method is used for the expansion of obstacle boundaries, and the Gauss pseudospectral method (GPM) is utilized to discretize this optimal control problem into a nonlinear programming (NLP) problem. In order to solve this NLP problem, sequential quadratic programming is applied. Considering that the GPM is insensitive to the initial guess, an online calculation method of vertical parking trajectory is proposed. In this approach, the offline vertical parking trajectory, which is calculated and stored in advance, is taken as the initial guess of the online calculation. The selection of an appropriate initial guess is based on the actual starting position of parking. A small parking lot is selected as the verification scenario of the AVP. In the validation of the algorithm, the parking trajectory planning is divided into two phases, which are simulated and analyzed. Simulation results show that the proposed algorithm is efficient in solving a parking trajectory planning problem. The online calculation time of the vertical parking trajectory is less than 2 s, which meets the real-time requirement.

## 1. Introduction

With the development of an intelligent transportation system (ITS), parking technology has gone through three stages: semi-autonomous parking, autonomous parking, and autonomous valet parking (AVP) [1]. The semi-autonomous parking system and autonomous parking system are used to help drivers park safely. Moreover, a shorter parking time can effectively reduce the negative impact of a parking process on road traffic. In addition, the last-mile transportation of autonomous driving can be achieved with an AVP system since users can send instructions through terminal devices to park or retrieve their cars [2]. In this context, parking technology is an indispensable part of the autonomous driving system.

Among the key technologies of AVP, researchers focus on the platforms, sensor setups, perception, environment model, maps, and motion planning [3]. As the basis for the realization of AVP, environmental perception is mainly realized by a fusion of ultrasonic radars and cameras [4]. On this basis, a parking guidance system (PGS) based on visual recognition is built, and environment information is shared between the vehicle and the infrastructure system [5]. Furthermore, the tracking control is utilized to move the vehicle along the planned target path by the lateral and longitudinal motion controls [6,7].

Considering that the effects of environment perception and tracking control mainly depend on the accuracy of the sensors, path planning plays an important role in the entire system as a decision-making part. Thus, vehicle path planning is a research hotspot in the intelligent AVP technology. In the early days, curves composed of straight lines and arc joints, like Dubins curve and Reeds–Shepp curve, were used to solve planning problems by setting the shortest path as the goal [8,9]. Additionally, the geometric construction method of a straight line combined with arc joints has been widely used in the path planning of parallel parking [10]. However, the curvature of the path is discontinuous despite that the path can be easily obtained by this method. Therefore, methods such as clothoid curve, B-spline, and polynomial curve are applied to optimize the curvature of the path, but these methods are difficult to apply in path planning with a complicated constraint model [11,12,13].

In subsequent development, the path planning algorithms of motion planning are mainly divided into the following four categories: graph-search, sample-based, interpolation, and numerical optimization [14]. Considering the simplicity of practical application, graph-search methods are more efficient than traditional path planning strategies [15]. For example, classical graph-search algorithms like the Dijkstra and A* algorithms are both widely used to search the shortest path [16,17,18]. Moreover, compared with the Dijkstra algorithm, heuristic guidance is combined with the A* algorithm to reduce search work. However, they are not efficient enough to obtain the shortest path in a complicated environment. On the contrary, both the Probabilistic Roadmap Method(PRM) and Rapid-exploration Random Tree(RRT), which are based on random sampling points for planning the best path in a space, can effectively deal with a path planning problem with complicated constraints [19,20]. The above methods are all effective in global planning. However, for local path planning, the artificial potential field (AFK) for obtaining the path along the direction of the field drop is more suitable [21].

Compared with the traditional algorithms mentioned above, heuristic algorithms (e.g., machine learning, neural network, fuzzy control, genetic algorithm, particle swarm optimization, and colony algorithm [22,23,24,25,26,27]) are more widely applied in solving path planning problems. In a complex dynamic environment, bio-inspired planning algorithms are effective for collision-free planning [28,29]. Additionally, in an actual urban environment, a motion planning method based on a layered strategy is more suitable for the application of autonomous driving [30]. Moreover, scholars tend to combine traditional algorithms with heuristic algorithms to obtain better planning results. For example, the model predictive control method is used for real-time path planning [31]. Furthermore, the path planning problem is described as an optimal control problem based on optimal control theory [32,33,34].

In general, the key to developing AVP technology is the cooperation between trajectory planning and parking management systems [35]. Problems such as complex environment models and low computational efficiency are common in existing trajectory planning methods. To solve these problems, a method that obtains the optimal trajectory of AVP by solving the optimal control problem is proposed. The vehicle kinematics model, obstacle avoidance constraints, and endpoint constraints are established. The shortest overall parking time and travel distance are set as the cost function of the optimal control problem, which are established to describe the parking trajectory planning problem. On this basis, the contribution of this paper has two main components: (i) A homotopic method is utilized to transform the obstacle avoidance constraint in each iteration, and the optimal solution is set as the initial guess of the next new problem. The purpose of this step is to increase the success rate of offline planning with less cost. (ii) An online calculation method of vertical parking trajectory is proposed, where the starting points are decided in the initial area of vertical parking to build a matrix. Additionally, the parking trajectory of each starting point is obtained by offline calculation and stored. To improve the real-time performance of online calculation, in the vertical parking phase, the appropriate trajectory data are selected as the initial guess of online calculation according to the actual starting position of parking. Then, the Gauss pseudospectral method (GPM) is used to obtain the vertical parking trajectory.

A small parking lot is selected as the scenario of AVP, and the parking trajectory planning is separated into two phases. In this context, the trajectories of the two phases are simulated and analyzed. On this basis, the vertical parking trajectory is calculated online to verify the effectiveness of the proposed algorithm.

The remainder of the paper is organized as follows. The AVP scenario and the optimal control problem for parking, including kinematics models and various constraints, are described in Section 2. The optimal control problem is discretized as the nonlinear programming (NLP) problem through the GPM, and the solution strategy of the NLP problem through the homotopic method is described in Section 3. The simulation results and discussion of parking trajectory are in Section 4, and an online planning method of vertical parking is proposed. Finally, the conclusion and future works are discussed in Section 5.

## 2. Trajectory Optimal Control Problem of AVP

### 2.1. AVP Scenario

**Figure 1:**
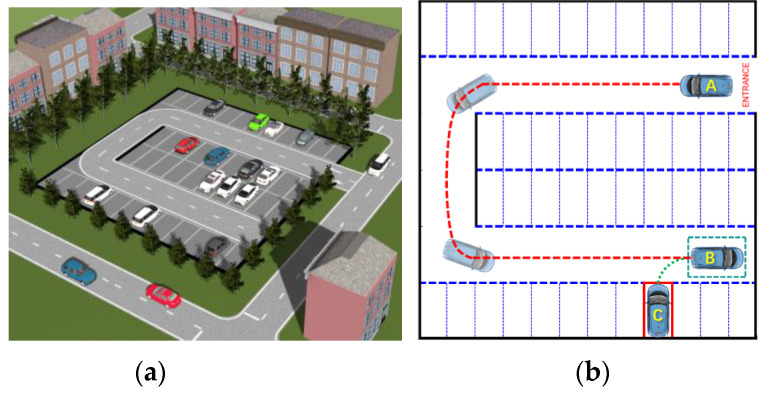
Parking lot schematic map: (**a**) parking lot in 3D view, (**b**) parking lot in 2D view.

In this paper, a small parking lot is selected as the scenario for verifying the algorithm. There is a U-shaped road in the parking lot, and the parking spaces on both sides of the road are vertical (see Figure 1a). Denoted by positions A, B, and C are the parking entrance, initial vertical parking position, and parking completion position, respectively. Based on the common parking process of a human driver, the parking process is divided into two phases: (i) The search for a vacant parking space, where the driver starts to seek a vacant parking space when entering the parking lot. Then the vehicle is moved to an initial parking area after finding a parking space. (ii) The vertical parking phase, where the vehicle moves backwards from the initial parking area until it is completely in the parking space. To facilitate parking trajectory planning, a 3D perspective of the parking space is set to top view (see Figure 1b). The initial vertical parking area and parking space are represented by the green dotted frame and the red solid frame, respectively, and the paths of the two phases are A–B and B–C.

Assuming that the parking lot is built with an infrastructure system, the vehicle can communicate with the system by Vehicle to Infrastructure (V2I) technology [36]. Moreover, vacant parking spaces can be detected by the system and allocated to vehicles entering the parking lot. In this context, the vehicle can be guided to the initial vertical parking area according to the system information. However, the guidance information provided by the system is not complete. Vehicle sensors, such as ultrasonic sensors, are needed to detect the environment for the accuracy of information around the vacant parking space. Besides, the parking trajectory in the vertical parking phase is obtained according to the initial parking position and the position relative to the vacant parking space. The trajectories of these two phases can be obtained in the following two means. One is that the trajectory is calculated by the server of the infrastructure system and sent to the vehicle, which is controlled to track the trajectory. Another is that the vehicle obtains part of the guidance information from the facility system, and then combines it with its own perception information for calculation. Also, the vehicle is controlled to track the obtained trajectory.

### 2.2. Kinematics Model

**Figure 2:**
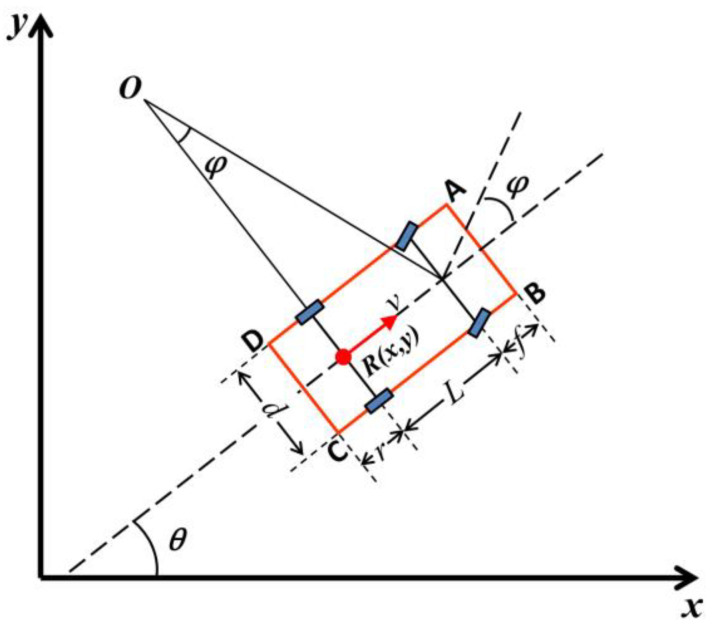
Kinematics model of vehicle.

According to Ackerman steering, all wheels are in a state of rolling, and the vehicle is not affected by lateral force during the parking process. Thus, the slip of the wheel is neglected, and the steering axis of the front wheel intersects the axis of the rear wheel. On this basis, a kinematics model of a vehicle in autonomous parking is built as shown in Figure 2, where *R*(*x*, *y*) denotes the center of the rear axle; *v* refers to the speed of the center of the rear axle; *O* stands for the center of the instantaneous turning of the vehicle; *f* and *r* denote the front suspension and rear suspension of the vehicle, respectively; *d* stands for the width of the vehicle; *L* is the wheelbase of the vehicle; *φ* stands for the equivalent front wheel swing angle; and *θ* refers to heading angle. Furthermore, A, B, C, and D are the four vertices of the vehicle body.

According to the vehicle kinematics model, kinematics differential equations can be obtained as follows:

(1) $$\begin{array}{c} \{\begin{array}{c} \dot{x}=v\left(t\right)\cdot \cos\theta \left(t\right)\\ \dot{y}=v\left(t\right)\cdot \sin\theta \left(t\right)\\ \dot{v}=a\left(t\right)\\ \dot{a}=j\left(t\right)\\ \dot{\theta }=v\left(t\right)\cdot \tan\varphi \left(t\right)/L\\ \dot{\varphi }=\omega \left(t\right) \end{array},\;t\in \left[t_{0,}t_{f}\right] \end{array}$$

where a and j refer to vehicle acceleration and jerk of the vehicle, ω denotes the angular velocity of the front wheel pendulum, and $t_{0}$ and $t_{f}$ stand for initial time and final time, respectively.

According to the geometric relationship, the coordinate values of A, B, C, and D can be obtained by the center point of the rear axle *R*(*x*, *y*) and are as follows:

(2) $$\begin{array}{c} \{\begin{array}{c} \left[Ax\left(t\right),Ay\left(t\right)\right]=\left(x\left(t\right)+\left(L+f\right)\cos\theta \left(t\right)-\frac{d}{2}\sin\theta \left(t\right),y\left(t\right)+\left(L+f\right)\sin\theta \left(t\right)+\frac{d}{2}\cos\theta \left(t\right)\right)\\ \left[Bx\left(t\right),By\left(t\right)\right]=\left(x\left(t\right)+\left(L+f\right)\cos\theta \left(t\right)+\frac{d}{2}\sin\theta \left(t\right),y\left(t\right)+\left(L+f\right)\sin\theta \left(t\right)-\frac{d}{2}\cos\theta \left(t\right)\right)\\ \left[Cx\left(t\right),Cy\left(t\right)\right]=\left(x\left(t\right)-r\cos\theta \left(t\right)+\frac{d}{2}\sin\theta \left(t\right),y-r\sin\theta \left(t\right)-\frac{d}{2}\cos\theta \left(t\right)\right)\\ \left[Dx\left(t\right),Dy\left(t\right)\right]=\left(x\left(t\right)-r\cos\theta \left(t\right)-\frac{d}{2}\sin\theta \left(t\right),y-r\sin\theta \left(t\right)+\frac{d}{2}\cos\theta \left(t\right)\right) \end{array} \end{array}$$

### 2.3. Physical Constraints

Considering safety and crew comfort, some state variables and control variables of the system are constrained as follows:

(3) $$\begin{array}{c} \{\begin{array}{c} \left|v\left(t\right)\right|\leq v_{max}\\ \left|a\left(t\right)\right|\leq a_{max}\\ \left|j\left(t\right)\right|\leq jerk_{max}\\ \left|\varphi \left(t\right)\right|\leq \varphi_{max}\\ \left|\omega \left(t\right)\right|\leq \omega_{max} \end{array},\;t\in \left[t_{0,}t_{f}\right] \end{array}$$

where $v_{max}$ stands for the maximum speed with $a_{max}$ for the maximum acceleration, $jerk_{max}$ denotes maximum acceleration change rate, $\varphi_{max}$ denotes the maximum front wheel swing angle, and $\omega_{max}$ is the maximum front wheel swing angle speed. Another purpose of constraining these variables is to make the planned trajectory as smooth as possible so that it will be relatively easier for each vehicle execution system (such as steering system, power system, braking system) to implement trajectory tracking.

### 2.4. Avoid Collision Constraints

**Figure 3:**
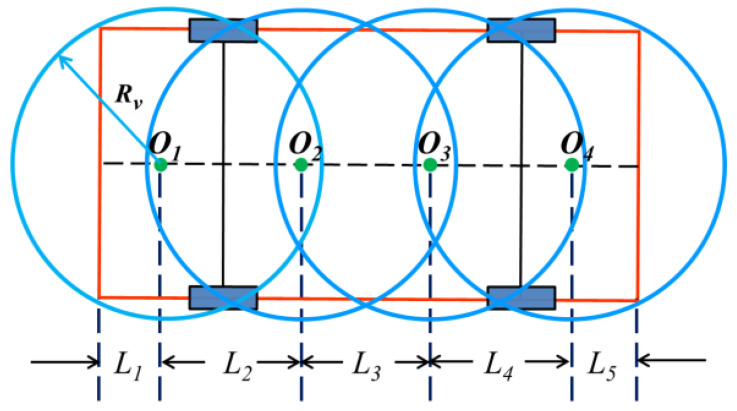
Circle position diagram of vehicle outside contour.

There is no collision allowed while the vehicle is parked. To this end, collision between vehicles and dynamic or static obstacles (e.g., other vehicles, pedestrians, road boundaries) must be considered. The vehicle contour is assumed to be rectangular. Considering the detection accuracy of on-board sensors and the detection blind area in practice, the four-circle model is selected to describe the vehicle contour in this paper, which is shown in Figure 3, where $O_{1}$, $O_{2}$, $O_{3}$, and $O_{4}$ denote the center of four vehicles. The geometric relationship is described as follows:

(4) $$\begin{array}{c} \{\begin{array}{c} L_{1}=L_{5}=\frac{1}{8}\left(r+L+f\right)\\ L_{2}=L_{3}=L_{4}=\frac{1}{4}\left(r+L+f\right)\\ R_{v}=\sqrt{L_{1}^{2}+{\left(d/2\right)}^{2}} \end{array} \end{array}$$

where $L_{1}$ stands for the distance from circle center $O_{1}$ to the rear of the vehicle, and $L_{5}$ stands for the distance from circle center $O_{4}$ to the front of the vehicle. Furthermore, $L_{2}$, $L_{3}$, and $L_{4}$ refer to the distance between $O_{1}O_{2}$, $O_{2}O_{3}$, and $O_{3}O_{4}$, respectively. $R_{v}$ denotes the radius of the outer circle.

From the above geometric relationships and the center point of the rear axle *R*(*x*, *y*), the positions of four centers are described as follows:

(5) $$\begin{array}{c} \begin{array}{c} \{\begin{array}{c} \left[O_{1}x\left(t\right),O_{1}y\left(t\right)\right]=\left(x\left(t\right)-\left(r-L_{1}\right)\cos\theta \left(t\right),y\left(t\right)-\left(r-L_{1}\right)\sin\theta \left(t\right)\right)\\ \left[O_{2}x\left(t\right),O_{2}y\left(t\right)\right]=\left(x\left(t\right)+\left(L_{1}+L_{2}-r\right)\cos\theta \left(t\right),y\left(t\right)+\left(L_{1}+L_{2}-r\right)\sin\theta \left(t\right)\right)\\ \left[O_{3}x\left(t\right),O_{3}y\left(t\right)\right]=\left(x\left(t\right)+\left(L+f-3L_{5}\right)\cos\theta \left(t\right),y\left(t\right)+\left(L+f-3L_{5}\right)\sin\theta \left(t\right)\right)\\ \left[O_{4}x\left(t\right),O_{4}y\left(t\right)\right]=\left(x\left(t\right)+\left(L+f-L_{5}\right)\cos\theta \left(t\right),y\left(t\right)+\left(L+f-L_{5}\right)\sin\theta \left(t\right)\right) \end{array},\;t\in \left[t_{0,}t_{f}\right] \end{array} \end{array}$$

**Figure 4:**
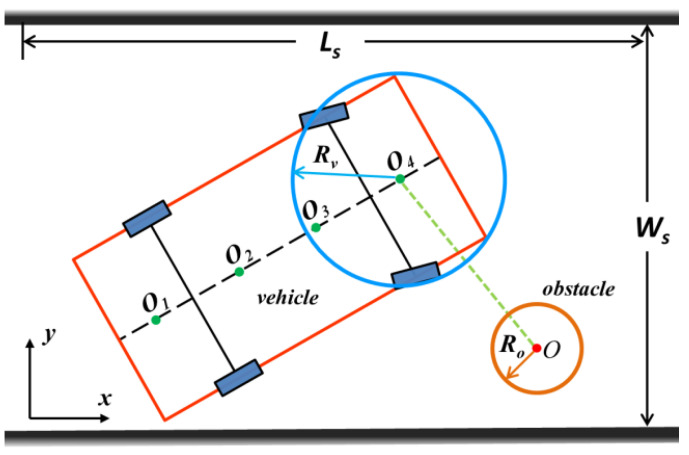
Schematic of avoidance between vehicle and obstacle.

Similarly, the contours of obstacles are described by multiple circumferential circles (see Figure 3). As shown in Figure 4, if the shapes of the obstacles are similar to a small square, such as pedestrians and trash cans, they can be described by a single circle for convenience. Therefore, the collision-free conditions between vehicles and obstacles can be described by the avoidance of the overlap between the circle of the vehicle contour and that of the obstacle contour. The strict mathematical description of security is shown in Equation (6):

(6) $$\begin{array}{c} {\left(O_{i}x\left(t\right)-Ox\left(t\right)\right)}^{2}+{\left(O_{i}y\left(t\right)-Oy\left(t\right)\right)}^{2}\geq {\left(R_{v}+R_{o}\right)}^{2} \end{array}$$

where O and $R_{o}$ denote the center and radius of the circumferential circle of the contour of the obstacle, respectively.

Otherwise, collision between a vehicle and road boundaries has to be considered. The movement of the vehicle is in an area bordered by a road boundary, where $L_{s}$ and $W_{s}$ denote the length and width of the road, respectively (see Figure 4). Hence, in the course of the movement, the four vertices of the vehicle, A, B, C, and D, all fall in the area expressed as follows:

(7) $$\{\begin{array}{c} \left[\mathrm{Ax}\left(t\right),\mathrm{Bx}\left(t\right),\mathrm{Cx}\left(t\right),\mathrm{Dx}\left(t\right)\right]\in \left(0,L_{s}\right)\\ \left[\mathrm{Ay}\left(t\right),\mathrm{By}\left(t\right),\mathrm{Cy}\left(t\right),\mathrm{Dy}\left(t\right)\right]\in \left(0,W_{s}\right) \end{array}$$

### 2.5. Terminal Conditions

**Figure 5:**
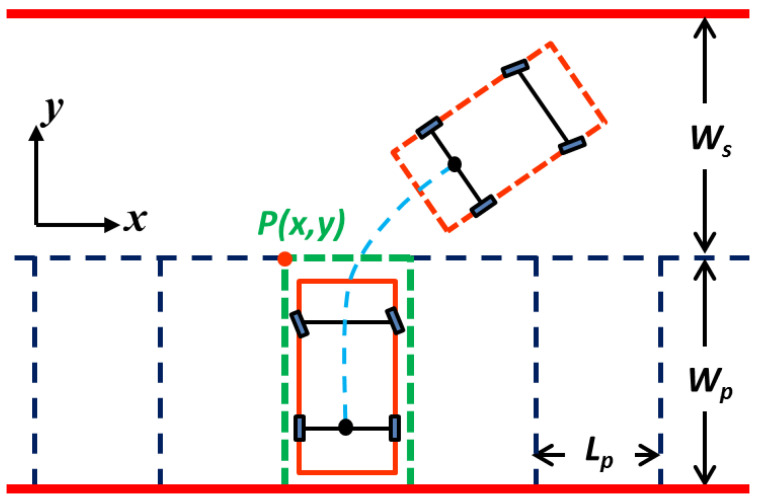
Schematic of the process of vertical parking.

The satisfaction of the above constraints ensures a collision-free motion of the vehicle from the starting moment $t_{0}$ to the ending moment $t_{f}$. Moreover, the terminal constraint of the vehicle at $t_{0}$ and $t_{f}$ is supposed to be taken into consideration. In this paper, the initial state and the end state of the vehicle are stationary, that is, $v\left(t_{0}\right)=0$, $v\left(t_{f}\right)=0$. Other states like acceleration at $t_{0}$ are selected depending on different scenarios. The four vertices of the vehicle must be inside the parking space at $t_{f}$. In addition, it is stipulated that the body of the vehicle should be parallel to the parking space as shown in Figure 5, where P(x,y) is the upper-left vertex of the vertical parking space. The terminal constraints at $t_{f}$ are as follows:

(8) $$\{\begin{array}{c} Px<\left[Ax\left(t_{f}\right),Bx\left(t_{f}\right),Cx\left(t_{f}\right),Dx\left(t_{f}\right)\right]<Px+L_{p}\\ -W_{p}<\left[Ay\left(t_{f}\right),By\left(t_{f}\right),Cy\left(t_{f}\right),Dy\left(t_{f}\right)\right]<Py\\ \theta \left(t_{f}\right)=\pi /2 \end{array}$$

where $L_{p}$ and $W_{p}$ denote the length and width of the parking space, respectively.

### 2.6. Trajectory Planning Optimal Control Problem

In this work, the minimum sum of $t_{f}$ and the driving distance are set as the cost function. Combined with the kinematics model, the collision avoidance constraints, the above-mentioned endpoint constraints, and the optimal control problem of autonomous parking trajectory planning are depicted as follows:

$$J=min\left(\omega_{1}\cdot t_{f}+\omega_{2}\cdot \int_{t_{0}}^{t_{f}}v\left(t\right)\mathrm{dt}\right)$$

(9) $$\begin{array}{c} s.t.\{\begin{array}{c} Differential\;equations\;of\;vehicle\;dynamics\;in\;Eq.\left(1\right)\\ Physical\;Constraints\;in\;Eq.\left(3\right)\\ Avoid\;collision\;constrains\;in\;Eqs.\left(4\right)\left(5\right)\left(6\right)\left(7\right)\\ Terminal\;constrains\;in\;Eq.\left(8\right) \end{array} \end{array}$$

where $\omega_{1}$ and $\omega_{2}$ are the weight of $t_{f}$ and the driving distance, respectively. When $\omega_{1}=1$ and $\omega_{2}=0$, only the optimal $t_{f}$ is considered in the cost function. In contrast, when $\omega_{1}=0$ and $\omega_{2}=1$, only the shortest driving distance is considered in the cost function. Furthermore, $\omega_{1}$ and $\omega_{2}$ are set to be 0.5.

## 3. Homotopic Method for Solving Optimal Control Problem

In Section 3, a unified trajectory optimization framework for the parking problem is presented. The GPM is utilized to discretize the above optimal control problem into a nonlinear programming problem for solving [37]. Here, the location of the collocation point is determined at first, and the state variables and control variables are discretized into unknown parameters at the collocation points. Then, Lagrange interpolation polynomials are constructed from these collocation points to determine the continuous state variables and control variables. By deriving the above Lagrange interpolation polynomials, the parking kinematics equation is transformed into algebraic equations at the collocation points, and the integral part of the cost function is discretized by the Gauss integral. Compared with the traditional collocation method and direct shooting method, the GPM, a kind of global collocation method, has better solving accuracy and convergence speed [38,39]. However, for nonsmooth problems, the convergence speed of the GPM is slower. To this end, a homotopic method is used to change the boundary of the obstacle avoidance constraint in the optimal control problem, and the solution of the last optimal control problem is used as the initial value of the next solution. The detailed framework is described as follows:

Step 1. Time domain transformation

The time domain of parking trajectory planning $t\in \left[t_{0},t_{f}\right]$ is transformed into a time domain of the GPM in [−1,1]:

(10) $$\tau =\frac{2t-t_{f}-t_{0}}{t_{f}-t_{0}},\;\tau \in \left[-1,1\right]$$

Step 2. Discretization of variables

Lagrange interpolation and Gauss quadrature equations are used to discretize the optimal control problem at a series of Legendre–Gauss (LG) points. The discrete points of the GPM include N+1 points composed of NLG points and $\tau_{0}=-1$. State variables and control variables are approximated at each configuration point as follows:

(11) $$\begin{array}{c} x\left(\tau \right)\approx X\left(\tau \right)=\overset{N}{\underset{i=0}{\sum }}L_{i}\left(\tau \right)\cdot X\left(\tau_{i}\right) \end{array}$$

(12) $$\mu \left(\tau \right)\approx U\left(\tau \right)=\overset{N}{\underset{i=0}{\sum }}L_{i}\left(\tau \right)\cdot U\left(\tau_{i}\right)$$

where x and μ stand for state variables and control variables, respectively. X and U refer to discrete variables, and $L_{i}\left(\tau \right)$ denotes Lagrange interpolation basis functions.

Step 3. Transformation of state equation and constraint equation

After the derivation of Equation (11), the obtained derivative of a state variable at collocation point $\tau =\tau_{k}$ can be approximated as follows:

(13) $$\begin{array}{c} \dot{x}\left(\tau_{k}\right)\approx \dot{X}\left(\tau_{k}\right)=\overset{N}{\underset{i=0}{\sum }}\dot{L_{i}}\left(\tau_{k}\right)\cdot X\left(\tau_{i}\right)=\overset{N}{\underset{i=0}{\sum }}D_{k,i}\cdot X\left(\tau_{i}\right) \end{array}$$

where D denotes a differential state matrix, which represents the differential value of the Lagrange interpolation basis function at each collocation point. Through Equation (13), a state equation can be transformed into equality constraints, as follows:

(14) $$\overset{N}{\underset{i=0}{\sum }}D_{k,i}\cdot X\left(\tau_{i}\right)-\frac{t_{f}-t_{0}}{2}f\left(X\left(\tau_{k}\right),U\left(\tau_{k}\right),\tau_{k}\right)=0$$

The obstacle avoidance constraints and endpoint constraints in parking trajectories are also discretized at the collocation points. The obtained equality constraints are as follows:

(15) $$\begin{array}{c} C\left(X\left(\tau_{k}\right),U\left(\tau_{k}\right),\tau_{k}\right)\leq 0 \end{array}$$

(16) $$E\left(X\left(\tau_{0}\right),\tau_{0,}X\left(\tau_{f}\right),\tau_{f}\right)=0$$

where C and E denote the obstacle avoidance constraint and endpoint constraint, respectively.

Step 4. Transformation of cost function

The integral term in the cost function can be approximated by the Gauss integral method. In this way, the cost function is transformed as follows:

(17) $$J=\Phi \left(X\left(t_{0}\right),t_{0},X\left(t_{f}\right),t_{f}\right)+\frac{t_{f}-t_{0}}{2}\overset{N}{\underset{i=0}{\sum }}\omega_{i}G\left(X\left(\tau_{i}\right),U\left(\tau_{i}\right),\tau_{i}\right)$$

where $\omega_{i}$ stands for the integral weight.

Step 5. The homotopic transformation of constraint equation

The optimal control problem of parking trajectory planning becomes unsmooth with the increase of environment complexity, which makes it difficult to solve the original problem by directly using the GPM. Thus, this paper proposes a homotopic method to improve the smoothness of the original problem. In the proposed method, the bounds of obstacle avoidance constraints in the original problem are taken as homotopic parameters to make the original problem of parking trajectory planning smoother. In this way, the unsmooth original problem is directly solved without changing the form of the original problem. In addition, the optimal solution is used as the initial value of the next calculation. To this end, Equation (15) is transformed as follows:

(18) $$\begin{array}{c} C\left(X\left(\tau_{k}\right),U\left(\tau_{k}\right),\tau_{k}\right)\leq H_{c} \end{array}$$

where $H_{c}$ denotes the homotopic parameter vector. To improve the convergence of the generated new problem, $H_{c}$ is added, releasing the constraint boundaries. Therefore, when $H_{c}$ evolves into 0, the problem becomes the original problem. Equation (18) can also be transformed as follows:

(19) $$\begin{array}{c} C\left(X\left(\tau_{k}\right),U\left(\tau_{k}\right),\tau_{k}\right)-H_{c}\leq 0 \end{array}$$

It is worth noting that Equation (19) has the same form as Equation (15). Therefore, the solution of the original problem can be obtained by constant iteration with a homotopic method. Moreover, the result of the last optimal control problem is taken as a better initial value, which reduces the difficulty of the next solution. As a result, the convergence speed of the solution is effectively improved.

**Figure 6:**
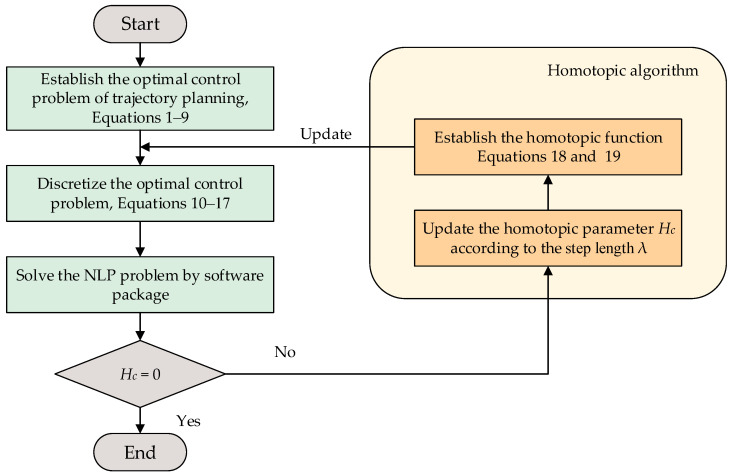
Flowchart of Gauss pseudospectral method (GPM)-based methodology for parking trajectory planning.

After the five steps above, the optimal control problem of parking trajectory planning is transformed into a discrete NLP problem by the GPM, and SNOPT is adopted as the software package in this paper. The overall flowchart of the proposed GPM-based methodology is demonstrated in Figure 6.

## 4. Simulation Results and Discussion

The trajectory planning of AVP is separated into two phases. Two obtained optimal trajectories of corresponding phases are spliced into a complete trajectory of the whole AVP. The code is executed on a laptop running Windows 7 with an Intel(R) Core(TM) i5-3210M 2.50 GHz processor and 8 GB RAM, written in MATLAB. The vehicle parameters and physical constraints used in the simulation are shown in Table 1.

### 4.1. Trajectory of Parking Space Searching Phase

In the first phase of the parking process, the vehicle moves from the entrance of the parking lot to the initial area of vertical parking. In this phase, it is assumed that there are no other dynamic obstacles, such as vehicles and pedestrians, but only some constraints of road boundary and parking space. The position of the vehicle at the entrance and the initial area of vertical parking are defined as follows:

(20) $$\begin{array}{c} \{\begin{array}{c} \left[Rx\left(t_{0}\right),Ry\left(t_{0}\right)\right]=\left(25.5,17.5\right)\\ 24\leq Ax\left(t_{f}\right),Bx\left(t_{f}\right),Cx\left(t_{f}\right),Dx\left(t_{f}\right)\leq 29\\ 0.5\leq Ay\left(t_{f}\right),By\left(t_{f}\right),Cy\left(t_{f}\right),Dy\left(t_{f}\right)\leq 4 \end{array} \end{array}$$

**Figure 7:**
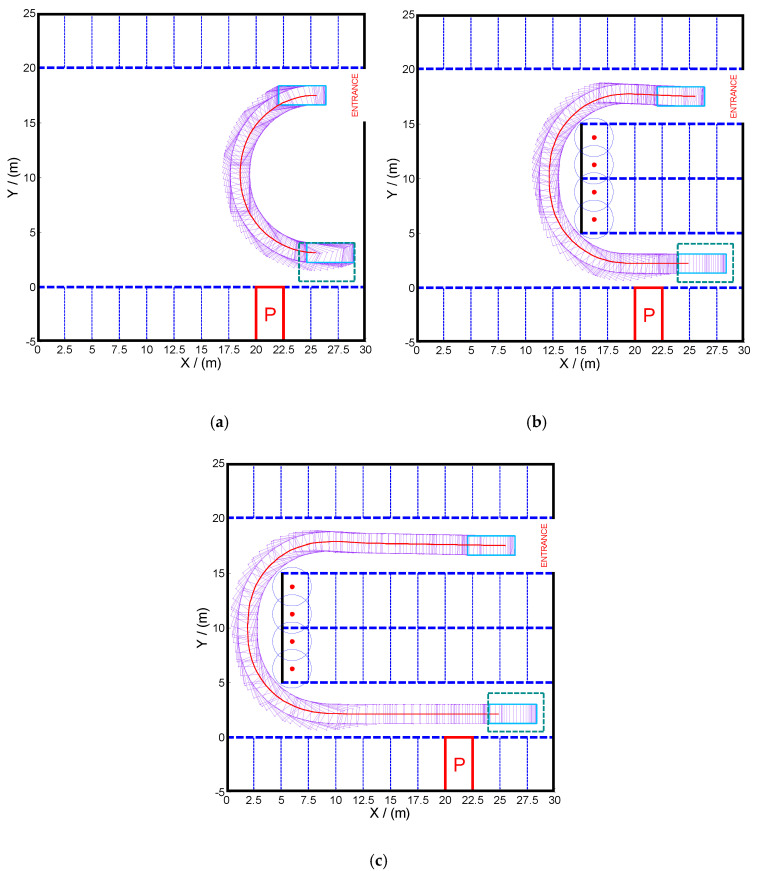
Trajectory planning in the parking space searching phase: (**a**) depicts the vehicle trajectory from the entrance of the parking lot to the initial area of vertical parking; (**b**,**c**) depict the vehicle trajectory under partial and complete constraints, respectively.

According to the above-mentioned algorithm, the trajectory planning problem of the first phase is solved without constraints, and the trajectory of the vehicle (see Figure 7a) is a circular arc. The termination condition is that all the vertices of the vehicle fall in the initial parking area. In this context, the vehicle reaches the top, rather than the middle, of the initial parking area to optimize the cost function, which is shown in Figure 7a. It is also found out that the trajectory intersects with the parking spaces in the middle of the parking lot, instead of those at the bottom or top of the parking lot. Therefore, by using the above-mentioned homotopic algorithm, the constraints of the middle parking spaces are gradually approximated to the constraints of the original optimal control problem. Then vehicle trajectories from the entrance to the initial area of parking are obtained by iterative solution, which are shown in Figure 7b,c.

### 4.2. Trajectory in Vertical Parking Phase

In the second phase of the parking process, the convenience of passengers in getting on and off has to be considered. To this end, the minimum width of the space on both sides of the vehicle when the parking ends is set to 0.3. Therefore, the minimum parking length min $L_{p}$ is set to 2.3, and the parking width $W_{p}$ is set to 5.0. Additionally, based on the range of the initial parking area, the road width $W_{s}$ is set to 4.5 (see Figure 5). For the convenience of calculation, the upper-left vertex of the vertical parking space is set as the origin of the coordinate system, that is, [Px,Py]=(0,0). Furthermore, the coordinate of the central point of the rear axle at the starting position is $\left[Rx\left(t_{0}\right),Ry\left(t_{0}\right)\right]=\left(5,1.5\right)$. In addition, the termination condition of parking completion is required to meet the constraints in Equation (8), and the equation of $Ry\left(t_{f}\right)=Lp/2$ to ensure that the vehicle is in the middle of the parking space at the ending moment.

**Figure 8:**
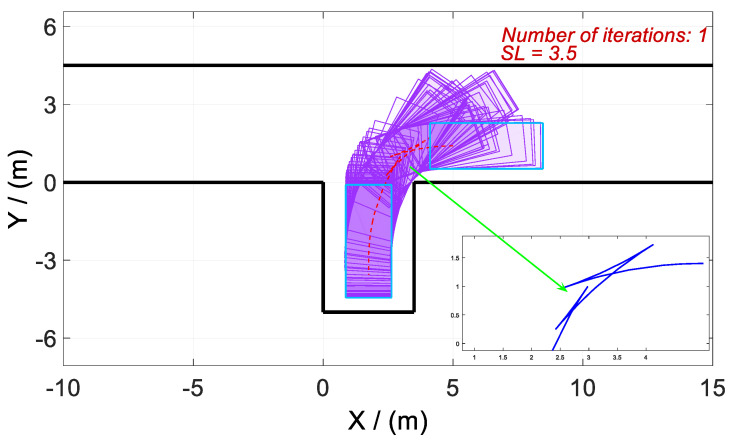
Optimized trajectories for Case 1 and $t_{f}=23.9753$ s.

**Figure 9:**
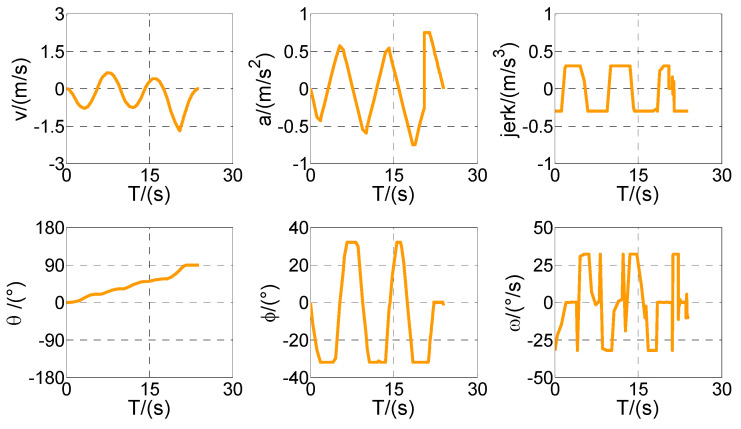
Optimized control and state variables in Case 1.

**Figure 10:**
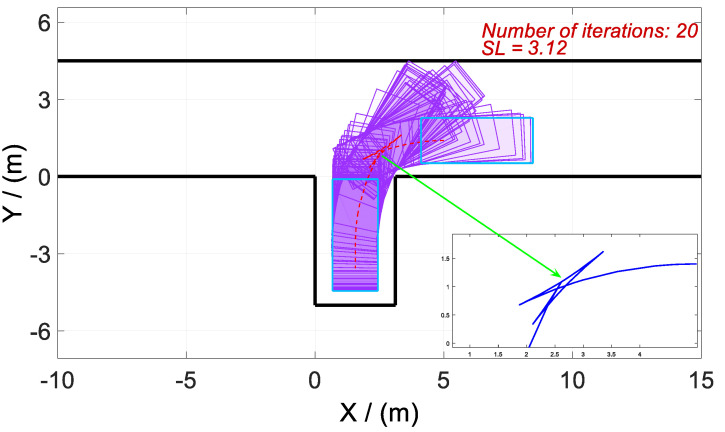
Optimized trajectories for Case 2 and $t_{f}=24.1324$ s.

**Figure 11:**
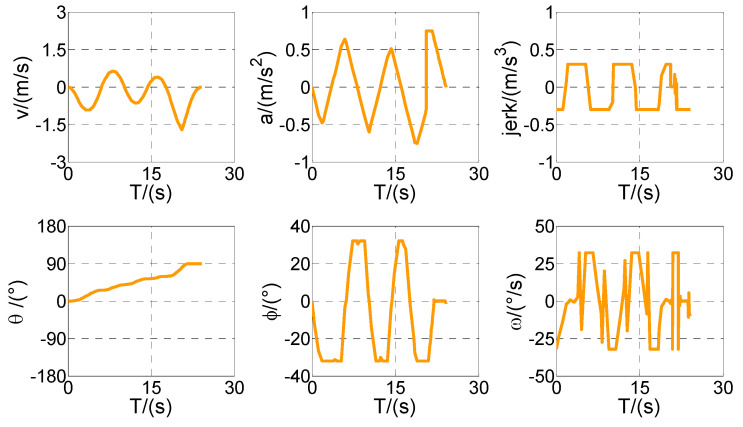
Optimized control and state variables in Case 2.

**Figure 12:**
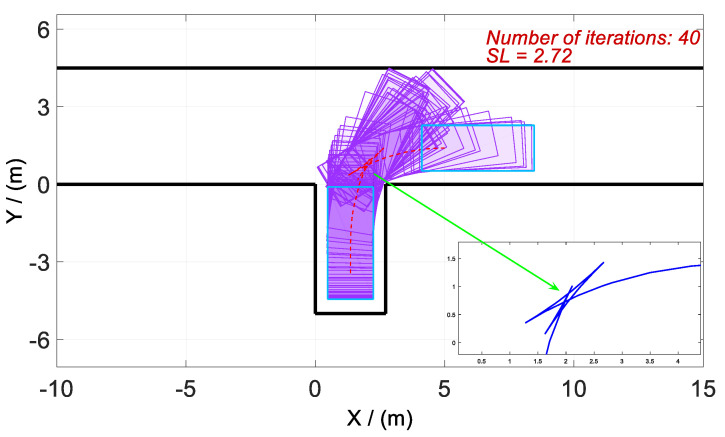
Optimized trajectories for Case 3 and $t_{f}=24.4173$ s.

**Figure 13:**
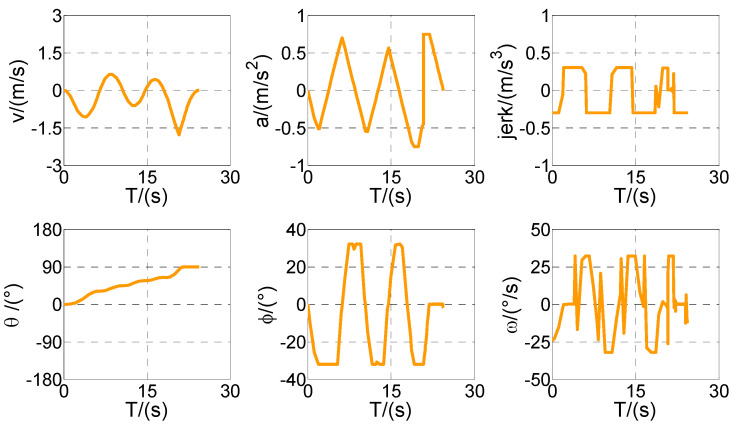
Optimized control and state variables in Case 3.

**Figure 14:**
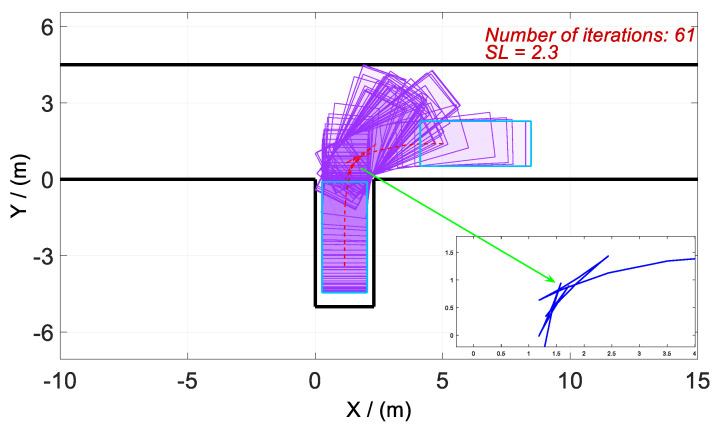
Optimized trajectories for Case 4 and $t_{f}=31.1608$ s.

**Figure 15:**
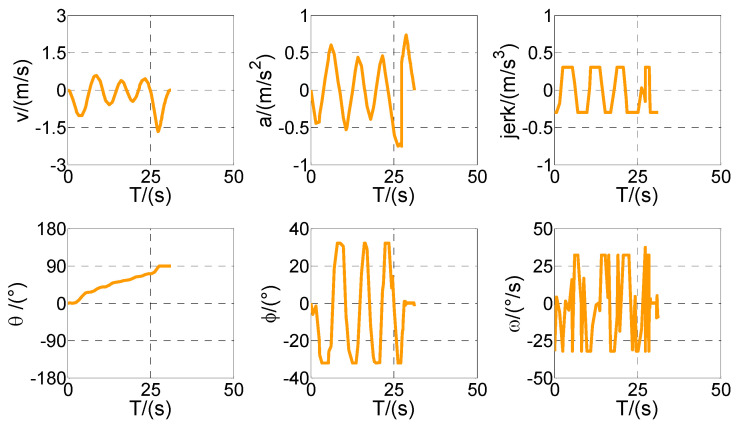
Optimized control and state variables in Case 4.

According to the above-mentioned homotopic algorithm, the parking space $L_{p}$ gradually decreases from 3.5 to the minimum value. In other words, the homotopic parameter $H_{c}=\left[3.5:\lambda :2.3\right]$, where the step length λ=0.02. Hence, the problem is solved with 61 times of iteration, and there is only a difference of *Lp* between each iteration process. Then, four vertical parking processes with different *Lp* values are selected for analysis and discussion. The values of *Lp* in Cases 1–4 are set as 3.50, 3.12, 2.72, and 2.30, respectively. The optimized trajectory, control variables, and state variables are illustrated in Figure 8, Figure 9, Figure 10, Figure 11, Figure 12, Figure 13, Figure 14 and Figure 15.

First of all, all vehicles can complete vertical parking and meet the above-mentioned termination constraints in Cases 1–4, which means that the vehicle is in the middle of the parking space when the parking ends. The control variables all present the feature of the bang–bang control (see Figure 9, Figure 11, Figure 13, and Figure 15) because time is required to be optimal in cost function. That is to say that the state variables are continuous and smooth despite that the control variables are oscillated.

As shown in Figure 8 and Figure 10, the trajectories in Cases 1 and 2 are similar. The adjustment of the vehicle occurs outside the parking space rather than inside, even if the parking space is relatively larger in Cases 1 and 2. This phenomenon is similar to our daily manual vertical parking process because the narrow parking space is not conducive to the adjustment of the vehicle posture.

As shown in Figure 10, Figure 11, Figure 12, Figure 13, Figure 14 and Figure 15, parking time $t_{f}$ shows a growing trend with the gradual decrease of *Lp*. Furthermore, the $t_{f}$ of Case 4 is the longest, and the reason is obvious. It is because of the narrow parking space resulting in six times of direction changes in Case 4, compared with only four times in Cases 1–3.

**Figure 16:**
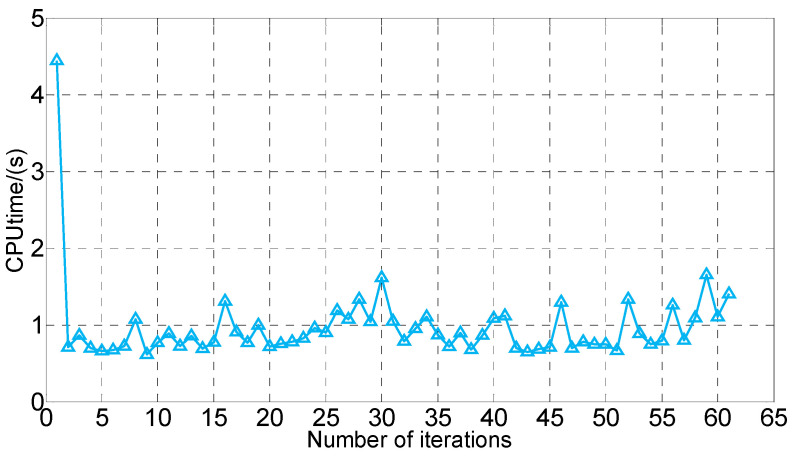
Computation time for each iteration with *Lp* from 3.5 to 2.3 m.

The traditional GPM takes a long time to directly solve the optimal parking problem, and even ends with nonconvergence when the parking space is narrow. Thus, this paper adopts a homotopic method to select a rational initial guess, which can solve this problem effectively. As depicted in Figure 16, the iteration time is less than 1 s in more than 90% of the cases. And the longest calculation time does not exceed 2 s. Longer iteration times occur at the beginning and end of the calculation for two reasons: (i) In the first iteration, the constraint is set to the calculation for the first time, and the increase of the constraint condition leads to the extension of the calculation time. (ii) At the end of the iteration, the rear space is narrow, so the process of trajectory optimization becomes difficult.

### 4.3. Online Computing Method for Vertical Parking Trajectory Planning

The offline calculation method of vertical parking trajectory planning is illustrated above. In the process of approaching the original optimal control problem by homotopic parameters, the overall calculation time is long even if the time cost of each iteration is short. At this time, real-time requirements will not be met. Thus, an online planning method of parking trajectory is proposed. Let us assume that the trajectories calculated by each iteration in the second phase are stored as a table. If the starting position and parking space size in practice are the same as in any case stored, the trajectory can be obtained directly from the stored trajectories by looking up the tables without any calculation. However, these ideal situations barely happen in practical applications. Considering this problem, the stored trajectory is used as an initial guess rather than an actual trajectory. The complete process is as follows: First, a parking space is found, and the vehicle is parked in its initial area. In this context, an optimal control problem for parking trajectory planning is formed. Then, the most similar set of trajectories is found from the stored data based on actual parking space size and the starting position, and used as the initial guess of the actual optimal control problem, which is solved by the GPM. The advantage is that the solving speed can be effectively improved by the proposed method. This is because the corresponding original problem of the found trajectory is similar to the current problem, which makes this trajectory similar to the current solution, being a good initial guess.

To verify the above method, a working condition with $L_{p}=2.5$, $\left[Rx\left(t_{0}\right),Ry\left(t_{0}\right)\right]=\left(5,1.5\right)$ is assumed, and its parking trajectory, used as the initial guess of the subsequent optimal control problem, is stored in the data table. Cases 5 and 6 are set up according to the distance from the actual parking starting position to point R. In each case, there are four vehicles whose start locations *R*’(*x*, *y*) are in the circles that take *R*(*x*, *y*) of the initial guess as the center and 0.2 and 0.5 as radii (as shown in Table 2). The GPM is also taken to solve the new optimal control problem for vertical parking trajectory planning.

**Figure 17:**
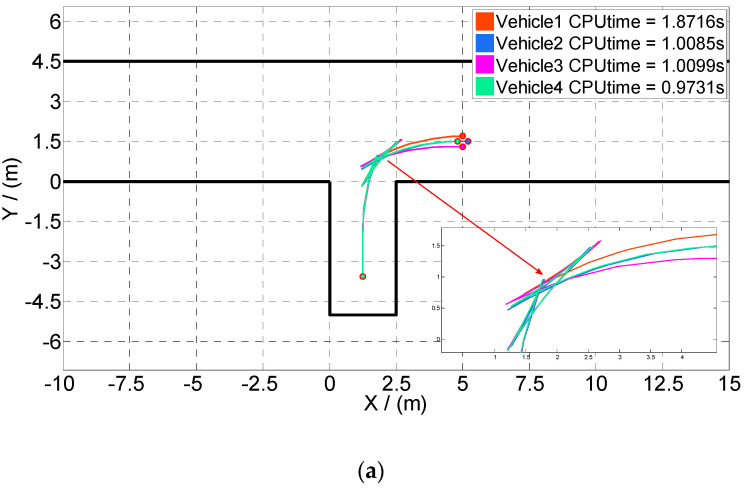

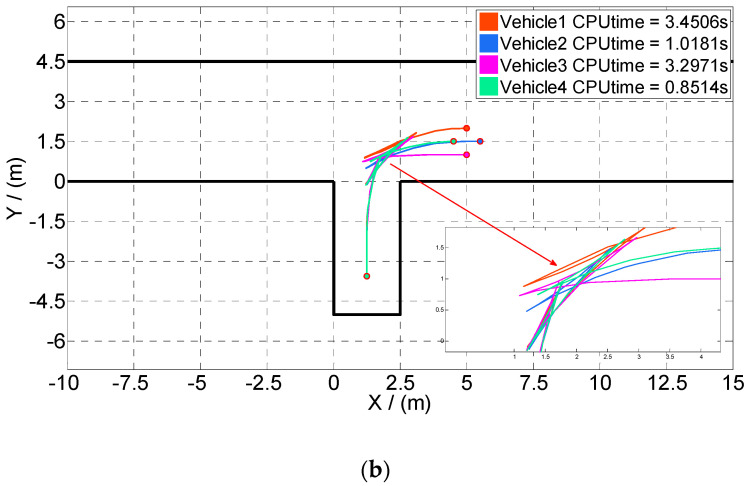
Optimal trajectory and calculation time obtained by online calculation of vertical parking. As depicted in Figure 17 (**a**) and (**b**), the starting points of vertical parking are in circles, taking the starting point of the initial guess as the center and 0.2 and 0.5 as radii.

As shown in Figure 17a,b, the new optimal control problems in Cases 5–6 can be solved quickly, and the solving speed of Vehicle 4 is the quickest, followed by that of Vehicle 2. For Vehicles 2 and 4, the vertical positions are the same as those in the initial guess, that is, $R^{\prime }y\left(t_{0}\right)=Ry\left(t_{0}\right)$, despite differences in the horizontal position. Therefore, their trajectories at the beginning phase of the parking are closer to the optimal ones. In contrast, the vertical positions of Vehicles 1 and 3 are different from those in the initial guess despite the same horizontal positions, which leads to a large deviation of the trajectories at the beginning phase. Therefore, the calculation time increases.

In Case 5, the maximal calculation time of all the vehicles is 1.87 s, which is acceptable in real-time parking applications. However, in Case 6, the calculation time of Vehicles 1 and 3 is more than 3 s. Comparing Case 5 and Case 6, it can be noted that the large deviation, especially horizontal deviation, of vehicle trajectory from the initial guess leads to a greater impact on calculation time. Therefore, the calculation time of the optimal control problem increases. In contrast, the deviation of the horizontal position has less effect on the calculation speed.

To verify the effectiveness and real-time performance of the proposed homotopic strategy, the calculation results of the two initialization methods are selected for comparison. The CPU time and *t_f_* of Cases 1–4 are recorded, and fail means nonconvergence in the calculation process. As presented in Table 3, the simulation costs of the proposed homotopic strategy are much lower than those of the other two strategies. Especially in conditions with small space (Cases 3 and 4), the calculation costs of the incremental strategy and the spatiotemporal decomposition strategy increase significantly, and the calculation may fail. We cite the following reasons to explain the excellent real-time performance of the proposed strategy: In the iterative solution process, only the width of the parking space changes according to the calculation step. Thus, optimal control problems of two adjacent calculations are very similar. In the homotopic strategy, the optimal solution of the previous calculation is used as the initial guess for each solution. And the calculation is guided by a high-quality initial guess. It is worth noting that since there is no initial guess stored when solving the problem of Case 1, the calculation time is slightly longer than that of other cases. This result is consistent with the conclusion in Figure 16.

As a summary, the processes of the online calculation of vertical parking trajectory are as follows: (i) A matrix, whose size is $R_{m\times n}$, is formed with starting points selected in the initial area of vertical parking. The optimal parking trajectories of these starting points are obtained by an offline method and stored. (ii) The starting point with the minimum deviation from the actual location is obtained by looking up the table of stored data. Note that the vertical deviation minimum takes higher priority than the horizontal one. Finally, the trajectory with the minimum deviation is taken as the initial guess of the optimal control problem. This optimal control problem is solved by the GPM, and an optimal trajectory of the actual parking is obtained.

## 5. Conclusions

In this paper, a method that combines the GPM and homotopic method to solve the optimal control problem is proposed for the trajectory planning of AVP. An optimal control problem is determined to describe the optimization problem of parking trajectory. Then the GPM is used to transform this optimal control problem into a nonlinear programming problem, which can be solved by sequence quadratic programming. The difficulty of solving is handled by a homotopic method, which adjusts the constraint boundary. The original problem is approached by iteration where the last solution result is taken as the initial value of the next solution. AVP trajectory planning is divided into two phases, and an online calculation method for the vertical parking phase is proposed. Furthermore, simulation results indicate that the proposed method can effectively improve the calculation speed and convergence of the solution. In the vertical parking phase, the online calculation time of trajectory planning is less than 2 s, which meets the real-time requirement.

In our future works, various sizes of parking scenarios will be taken into account. Furthermore, we will study the effective solving of the multivehicle coordinated trajectory of connected and automated vehicles in a parking lot.

## Figures and Tables

**Table 1:** Vehicle parameters and physical constraints.

Parameter	Description	Value
L	Wheelbase	2.560 m
f	Front overhang	0.902 m
r	Rear overhang	0.883 m
d	Width	1.765 m
$v_{max}$	Maximum speed	3.0 m/s
$a_{max}$	Maximum acceleration	0.75 m/s^2^
$jerk_{max}$	Maximum acceleration rate of change	0.3 m/s^3^
$\varphi_{max}$	Maximum front wheel swing angle	0.56 rad
$\omega_{max}$	Maximum front wheel swing angular velocity	0.56 rad/s

**Table 2:** Actual parking start position.

ID	Case 5		Case 6	
	$R^{\prime }x\left(t_{0}\right)$	$R^{\prime }y\left(t_{0}\right)$	$R^{\prime }x\left(t_{0}\right)$	$R^{\prime }y\left(t_{0}\right)$
Vehicle 1	5.0	1.7	5.0	2.0
Vehicle 2	5.2	1.5	5.5	1.5
Vehicle 3	5.0	1.3	5.0	1.0
Vehicle 4	4.8	1.5	4.5	1.5

**Table 3:** Comparison of the performances of distinct strategies.

	Incremental Strategy [40]		Spatiotemporal Decomposition [41]		Homotopic Strategy	
	CPU Time	$t_{f}$	CPU Time	$t_{f}$	CPU Time	$t_{f}$
Case 1	47.2232	22.4859	41.9217	25.5481	4.4415	23.9753
Case 2	42.9378	37.6651	65.5836	27.6475	0.7151	24.1324
Case 3	87.6384	fail	89.3382	28.8263	1.0877	24.4173
Case 4	115.4827	fail	92.7271	31.0379	1.4082	31.1608
